# The challenges of contemporary wound care

**DOI:** 10.1186/1757-1146-4-S1-I6

**Published:** 2011-05-20

**Authors:** Bill McGuiness

**Affiliations:** 1 La Trobe University Alfred Health Clinical School, La Trobe University, Melbourne, Vic, 3181, Australia President, Australian Wound Management Association

## 

Providing effective wound care to clients within the Australian context poses a number of challenges. For the clinician keeping abreast of continually changing arrays of products and supporting evidence is often daunting. For the client making sense of often conflicting advice and managing the personal costs of their treatment poses a significant challenge. For the health care provider providing efficient wound care within budgetary constraints is often difficult. Each of the above perspective is examined and practical solutions discussed in order to help improve contemporary wound management practices. Approaches to categorising wound management products will be presented as well as a list of user friendly resources that can be used by the clinician to judge their efficacy. Contemporary wound management techniques will be discussed with a focus on simple interventions that will help reduce delayed wound healing. The community awareness campaign being conducted by The Australian Wound Management Association will also be presented providing clinicians with possible avenues for further involvement.

